# Global sea level change signatures observed by GRACE satellite gravimetry

**DOI:** 10.1038/s41598-018-31972-8

**Published:** 2018-09-10

**Authors:** Taehwan Jeon, Ki-Weon Seo, Kookhyoun Youm, Jianli Chen, Clark R. Wilson

**Affiliations:** 1 Department of Earth Science Education, Seoul National University, Seoul, 08826 Republic of Korea; 2 Center for Space Research, University of Texas at Austin, Austin, Texas 78759 USA; 3 Department of Geological Sciences, Jackson School of Geosciences, University of Texas at Austin, Austin, Texas 78712 USA

## Abstract

Ice mass loss on land results in sea level rise, but its rate varies regionally due to gravitational self-attraction effects. Observing regional sea level rates by ocean mass change using the Gravity Recovery and Climate Experiment (GRACE) gravity solutions is difficult due to GRACE’s spatial resolution (~a few hundred km) and other limitations. Here we estimate regional sea level mass change using GRACE data (without contributions from temperature and salinity variations) by addressing these limitations: restoring spatially spread and attenuated signals in post-processed GRACE data; constraining ocean mass distribution to conform to the changing geoid; and judging specific corrections applied to GRACE data including a new geocenter estimate. The estimated global sea level mass trend for 2003–2014 is 2.14 ± 0.12 mm/yr. Regional trends differ considerably among ocean basins, ranging from −0.5 mm/yr in the Arctic to about 2.4 mm/yr in the Indian and South Atlantic Oceans.

## Introduction

Variations of sea level reflect both ocean mass and steric changes^1^. The former is associated with terrestrial ice and water mass exchange with the oceans, and the latter includes volumetric variations associated largely with thermal expansion and to a lesser extent salinity change. One of the promises of satellite gravity observations of Earth, realized through the GRACE mission, has been to observe Global Mean Sea Level (GMSL) rise associated with ocean mass increase^2^. As a first approximation, the spatial distribution of ocean mass change can be considered uniform, having the opposite sign of terrestrial water and ice mass storage changes. However, the ocean surface is nearly an equipotential, conforming on average to the geoid, whose shape is governed by Earth’s gravity field. The gravity field changes as variable ocean mass is distributed into irregularly shaped basins, continental water and ice storage varies at diverse locations, and Earth’s mass is redistributed due to the varying load. The sea level equation was developed to describe these mass effects, including self-attraction, on sea level change^3^. Subsequently, the theory has been refined to include the changing area of the oceans due to shoreline migration and Earth’s rotational feedback^4^. Many studies have used these results to predict sea level change due to specific water and ice mass changes^5–7^. These changes also have been called sea level fingerprints^5^, and are denoted by Δ*h*_s_ in this paper.

The GRACE mission has provided direct observations of ocean mass change on a global scale, but regional change estimates have not been directly compared with Δ*h*_s_ due to limitations of GRACE observations associated with the spatial resolution and the uncertainties in spatially low-frequency signals. A recent study found that Δ*h*_s_ over large ocean basins, showed annual phase in agreement with *in-situ* ocean bottom pressure observations, but magnitudes were only slightly different from GMSL change^8^. Difficulties in observing regional changes are due to the limited spatial resolution of GRACE. This causes the gravity signal of water and ice storage changes over land (with magnitudes of many centimetres of water) to ‘leak’ into adjacent ocean regions, where magnitudes are only a few millimetres. A number of attempts have been made to suppress this leakage problem. Mascon solutions, for example, have provided spatially gridded data with significantly reduced signal leakages by effectively separating signal between land and oceans^9–11^. However, a recent study showed that mascon data have unrealistic signals present along the coastline of Greenland^12^, indicating that this approach to leakage correction is imperfect. An effective way to correct this leakage problem is an algorithm called forward modelling^13^. The algorithm has been used in a number of studies to estimate mass changes that are consistent with GRACE observations and constrained by coastline geography, with uniform mass distribution over the oceans. Here we modified the algorithm by enforcing gravitationally consistent Δ*h*_s_ in place of uniform ocean mass distribution as attempted in the previous studies^14,15^. To calculate Δ*h*_s_, we simplified the modern form of the sea level equation, considering that our main purpose is to compare its predictions with monthly GRACE ocean signals, as described below. Simplifications included taking the area of the ocean as constant (neglecting shoreline migration) since Δ*h*_s_ is being evaluated over a short period of time (~12 years). Further, since the rotational feedback effect is nominally corrected in GRACE data^16^, the contribution is not included in our Δ*h*_s_ solution. Additionally we assume Earth’s response to surface load changes over the study period to be elastic, and that time-variable geoid height changes due to ice age effects are removed by the full Stokes spectrum of Post-Glacial Rebound (PGR) models.

Mass changes due to terrestrial water storage, ice sheet, and glacier changes were obtained by the forward modelling (FM) algorithm (hereafter called FM solutions). FM solutions, as refined GRACE solutions, yield two useful fields. One is Δ*h*_s_ itself, and the other is the estimated leakage of terrestrial water and ice storage changes into the GRACE signal over the oceans (see Methods). By subtracting this leakage from the GRACE data, we obtain a leakage-corrected GRACE ocean signal (denoted as Δ*h*_g_) that is mostly free of leakage from land, and more importantly, ought to conform approximately to changes in the geoid.

However, Δ*h*_g_ will not conform exactly to the geoid because it retains residual effects of ocean dynamics, atmospheric pressure, and noise, although these are largely corrected using geophysical models and filtering (see Methods for more details on the data processing). These residual effects tend to have relatively small spatial scales compared to geoid changes, so we can effectively estimate regional sea level (mass) changes associated with the geoid change by averaging over larger scale ocean basins. We needed to choose ocean basin sizes which would minimize the contribution of smaller-scale residual effects, in order to reveal larger spatial scales associated with geoid changes. Thus, larger scale errors are not suppressed by basin-scale averaging, and could affect both Δ*h*_s_ and Δ*h*_g_. Then, differences between ocean basin averages of Δ*h*_s_ and Δ*h*_g_ would be an indication that larger spatial scale errors are present. Therefore we use differences between area-weighted ocean basin averages of Δ*h*_s_ and Δ*h*_g_, as a self-consistency test to judge among various choices in standard GRACE processing steps. We then identify which choices yield the most self-consistent results, and use these to estimate regional and global ocean mass change.

There are many choices among models and methods in GRACE data processing that can influence estimates of GMSL mass changes. For example, GRACE Tellus (http://grace.jpl.nasa.gov) recommends the replacement of GRACE degree-2 and order-0 spherical harmonics (SH) coefficients with Satellite Laser Ranging (SLR) values^17^. For the correction of the PGR effect, the website also recommends use of the PGR model of A *et al*.^18^ based on ICE-5G (VM2)^19^ to remove that contribution. We evaluate these and other GRACE data processing choices using self-consistency to judge the best, in the sense of making Δ*h*_s_ and Δ*h*_g_ most similar. We consider three standard GRACE data processing steps: (a) substitutions for degree-2 SH coefficients in GRACE solutions, (b) choice of a PGR model, and (c) estimation of SH degree-1 (geocenter) terms. Since GRACE data do not contain degree-1 terms, we first consider (a) and (b) to find the most self-consistent choices based on the degree-2 terms and higher, and then address (c).

Questions about the quality of GRACE degree-2 SH coefficients have been raised in previous studies^17,20^, and it has become common to substitute SLR measurements of ΔC_20_ in place of GRACE coefficients^17^. An alternative is to retain GRACE ΔC_20_ coefficients, but correct them for known contamination (aliasing) from S_2_ and K_2_ ocean tides^21^. Similarly, GRACE ΔC_21_ and ΔS_21_ coefficients might be used as they are (recognizing that they retain significant long-period pole tide contamination^16^), or they might be modified as described by Wahr *et al*.^16^, or replaced entirely using estimates derived from polar motion^20^. Finally, although there are a number of published PGR models, we evaluate just three from the recent literature, those of A *et al*.^18^, Peltier *et al*.^22^, and Purcell *et al*.^23^.

**Figure 1:**
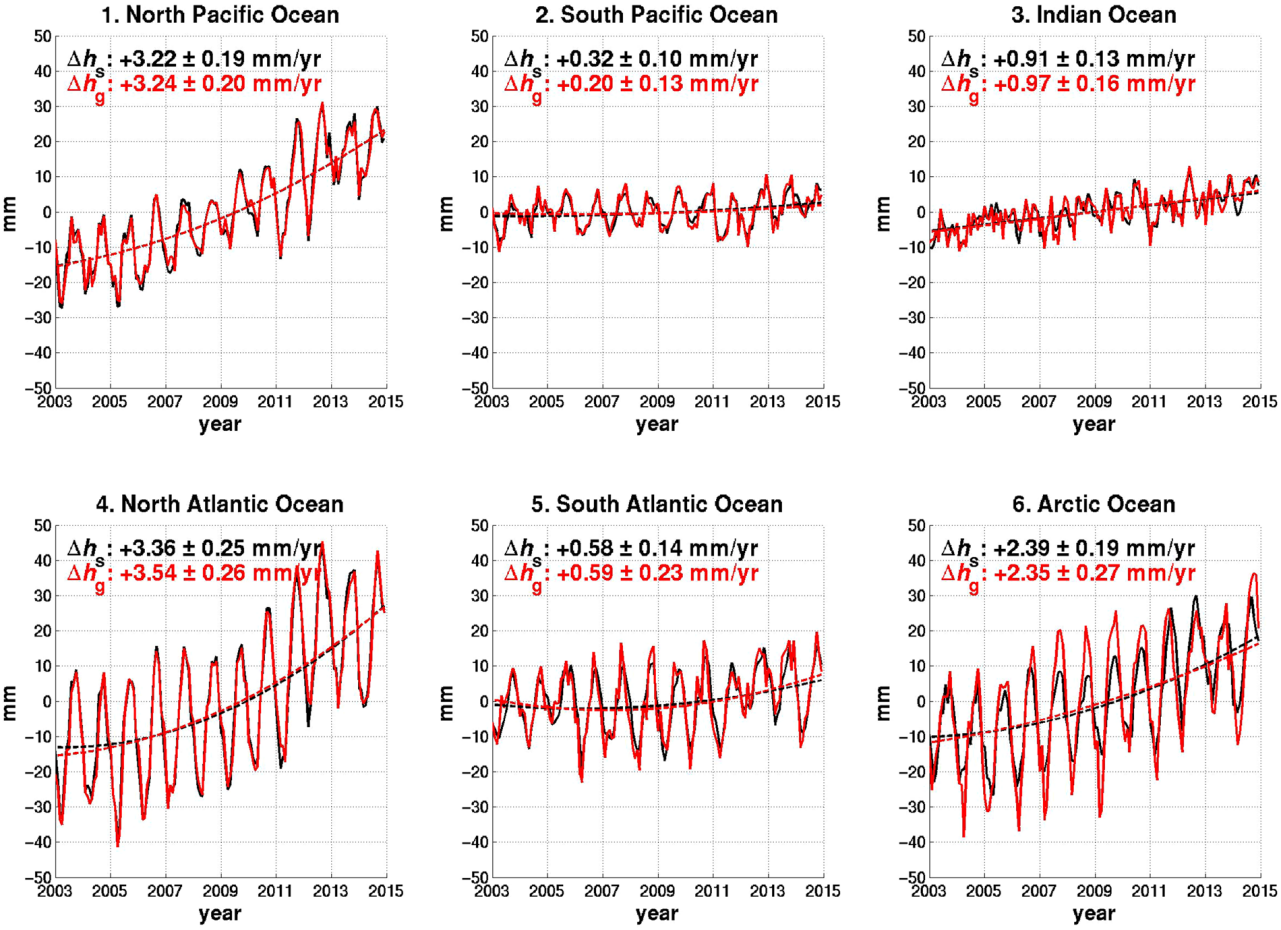
Self-consistency of GRACE data post-processed by the preferred methods. Smoothed Δ*h*_s_ (black solid line) and Δ*h*_g_ (red solid line) of sea level (mass) variation in millimetres per year over 6 ocean basins for January 2003 to December 2014. Estimates use the ICE-6G PGR model by Peltier *et al*.^22^. ΔC_20_ are GRACE estimates with S_2_ and K_2_ aliasing corrections, and ΔC_21_ and ΔS_21_ are estimates from polar motion. Degree-1 SH coefficients have not been included. Trends are estimated from second-order polynomial least square fits after removing seasonal variations, and the uncertainties are given at 2σ (95%) confidence level.

With three possible ways to adjust or substitute for ΔC_20,_ three for ΔC_21_ and ΔS_21_, and three PGR model choices, there are altogether 27 different combinations. Using monthly GRACE solutions provided by Center for Space Research (CSR) and GeoForschungsZentrum in Potsdam (GFZ), we examined all combinations, with full details given in the Methods section. The preferred choices that emerged as most self-consistent for CSR solutions use CSR GRACE ΔC_20_ with corrections for S_2_ and K_2_ ocean tide aliasing (removing periods of 161 days and 3.74 years, respectively^24^); substitute polar motion estimates of ΔC_21_ and ΔS_21_ in place of GRACE values; and adopt the Peltier *et al*.^22^ model in place of that from A *et al*.^18^. GFZ solutions show good self-consistency with different combination of processing methods (use of SLR ΔC_20_, adjusted GRACE ΔC_21_ and ΔS_21_ values from Wahr *et al*.^16^, and PGR model by Peltier *et al*.^22^), but overall performance is lower than the most self-consistent choices using CSR solutions (Supplementary Figs S6 and S7). This is mostly because GFZ ΔC_20_ is significantly corrupted by spurious long-term variations, so self-consistency is low when GFZ ΔC_20_ is incorporated. Both Δ*h*_s_ and Δ*h*_g_ time series, based on the CSR solution with the preferred methods, are shown in Fig. 1 for individual basins. They agree well with one another in six major ocean basins considered.

**Figure 2:**
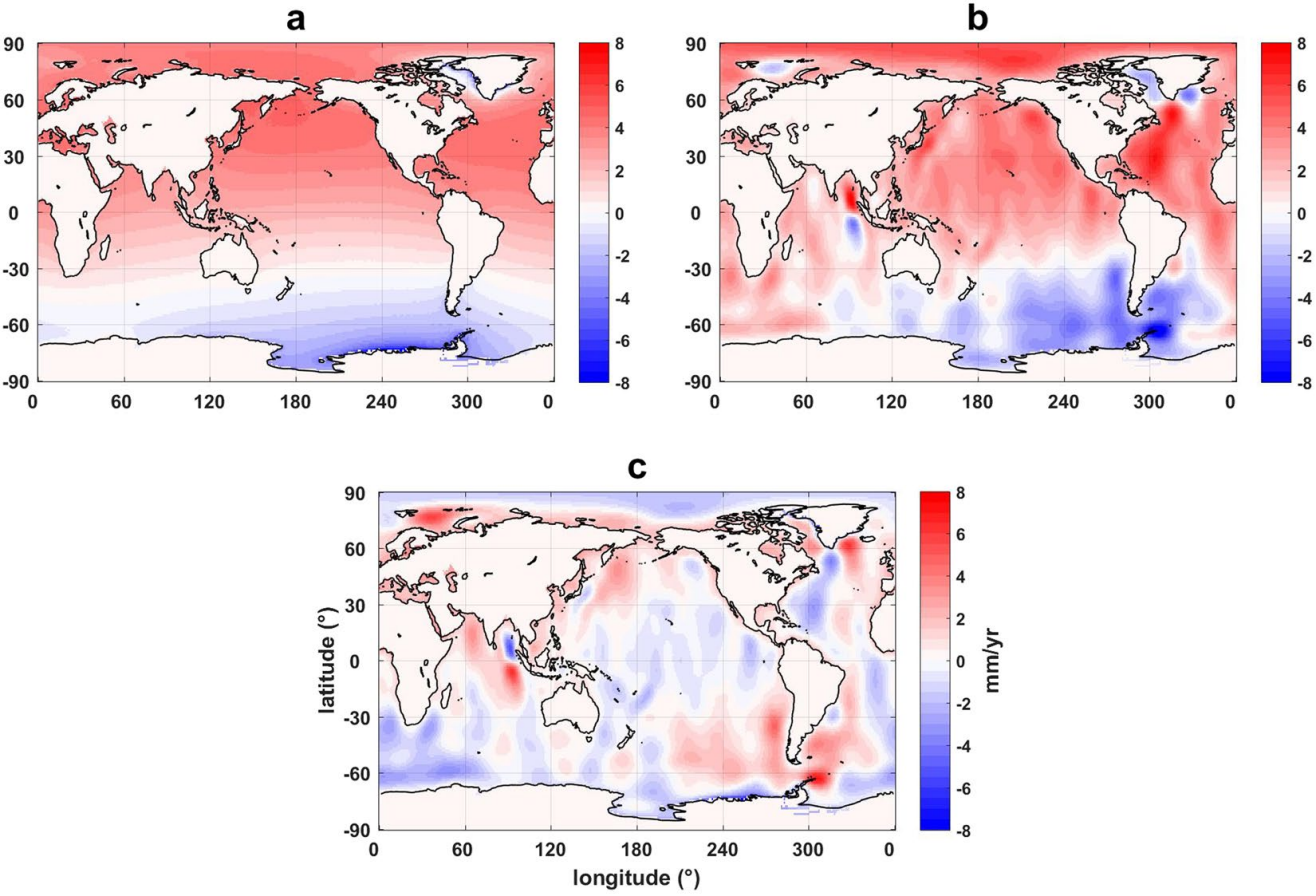
Trend map of Δ*h*_s_ and Δ*h*_g_ for the most self-consistent CSR GRACE data. (**a**) Linear trend map of Δ*h*_s_. (**b**) Linear trend map of Δ*h*_g_. (**c**) The difference of trend maps of Δ*h*_s_ and Δ*h*_g_. The results shown here are based on the preferred models and methods identified through consistency checks, without the contribution of degree-1 (geocenter) changes. Estimates use the ICE-6G PGR model of Peltier *et al*.^22^, ΔC_20_ GRACE estimates with S_2_ and K_2_ aliasing corrections, and ΔC_21_ and ΔS_21_ estimates from polar motion. Trends are estimated from second-order polynomial least square fits after removing seasonal variations in the study period (2003–2014).

Figure 2 shows linear rate maps of the Δ*h*_s_ and Δ*h*_g_, from a least square linear fit to time series at every 1 × 1 degree grid point for the period 2003–2014. The preferred models and methods based on CSR solutions described above are used to create these maps. Large scale features of ocean mass rate are clearly similar, and the leakage from land is almost completely removed from Δ*h*_g_ (Fig. 2b). However, changes at smaller spatial scales in Fig. 2b (Δ*h*_g_) are not present in Fig. 2a (Δ*h*_s_). As mentioned above, the difference between two solutions (Fig. 2c) can be attributed to residual errors in GRACE data. For example, residuals of north-south stripe are still visible even after using de-striping filters, and there are other differences associated with ocean dynamics (*e*.*g*. in the western Pacific Ocean and Atlantic Ocean)^25^ and post-seismic deformation (near Sumatra and Japan)^26^. It is also notable that a spatial signature of SH degree-2 and order-1 dominates the difference map. This feature would be associated with un-modelled PGR effect and/or rotational feedback^27^.

**Figure 3:**
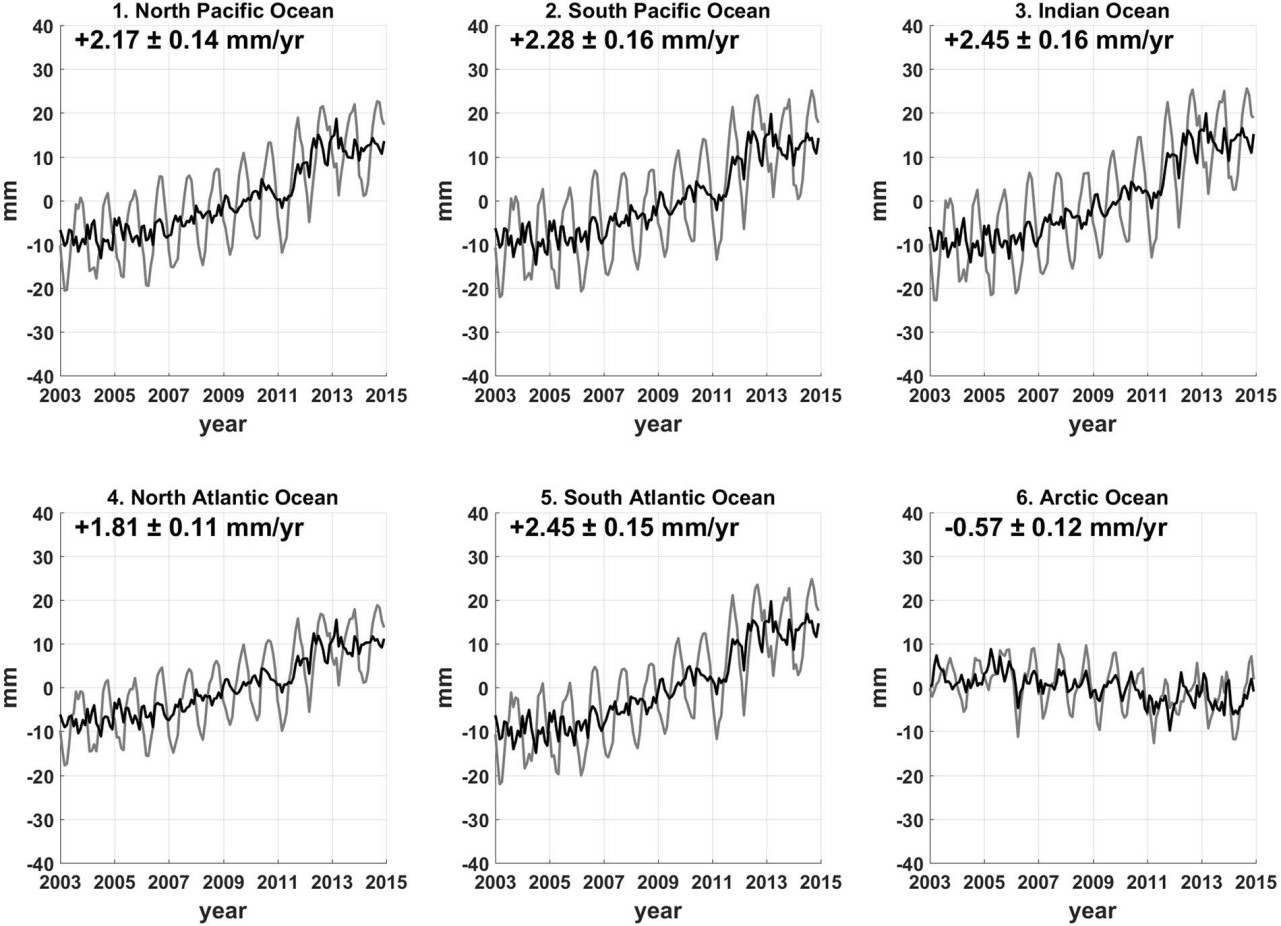
Mass contribution of sea level change with the contribution of degree-1 estimates. Sea level changes are examined by Δ*h*_s_ of the most self-consistent GRACE data with the contribution of degree-1 estimates from this study, shown in the 6 major ocean basins from January 2003 to December 2014. The gray lines are summations of black curves in Fig. 1 and contribution of degree-1 variation. Black lines represent time series with annual cycles removed from grey lines, and the trends and uncertainties shown in the figure are estimated from black time series by using second-order polynomial fitting with 2σ confidence level. In contrast to the others, sea level change in the Arctic shows a definite decreasing trend.

**Figure 4:**
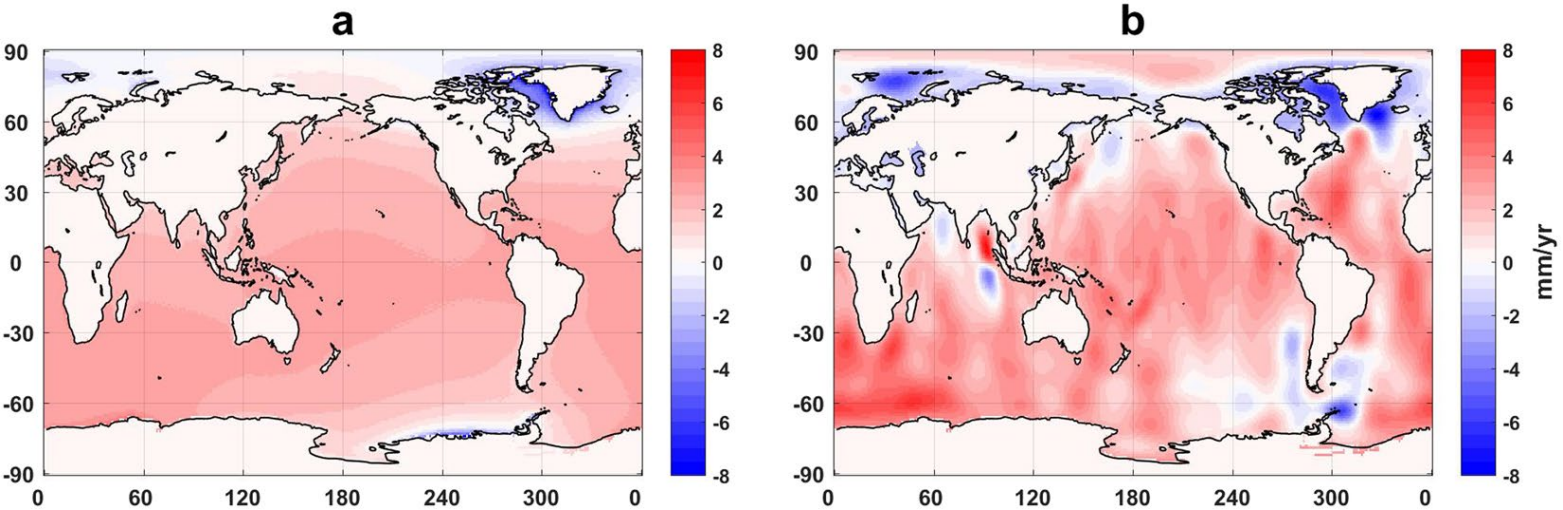
Trend map of Δ*h*_s_ and Δ*h*_g_ with the contribution of degree-1 estimates. Complete trend map of Δ*h*_s_ (**a**) and Δ*h*_g_ (**b**) from January 2003 to December 2014, adding the contribution of degree-1 estimates to the results displayed in Fig. 2. Both are derived from the preferred models and methods as discussed in the text.

We now consider how to estimate SH degree-1 (geocenter) changes. These are not available in GRACE solutions (given in a centre of mass (CM) reference frame), but are important in obtaining an ocean mass rate. Swenson *et al*.^28^ developed an algorithm for estimating geocenter change from GRACE data, but the estimates can be revised by incorporating properly corrected GRACE data. We examined an alternative degree-1 estimate based on the forward-modelling output over land and Δ*h*_s_ over the oceans (see Methods). We confirm the superior performance of this approach in a synthetic data test (Supplementary Fig. S8) compared to the original method. We applied this modified method to real GRACE data which had been processed using the preferred degree-2 substitutions and PGR correction as judged by the self-consistency test. Our degree-1 estimates add about 0.41 ± 0.03 mm/yr to mean ocean mass rate from 2003 to 2014, significantly larger than the value of Swenson *et al*.^28^ (~0.16 mm/yr). Figure 3 shows time series of Δ*h*_s_ in the 6 ocean basins after including geocenter changes, and these can be compared with black curves in Fig. 1. Global ocean mass rate maps are shown in Fig. 4 for Δ*h*_s_ and Δ*h*_g_ after inclusion of geocenter changes, which can be compared with Fig. 2.

Including the geocenter contribution, the total ocean mass rate for 2003–2014 is 2.14 ± 0.12 mm/yr (uncertainties given at 2σ confidence level). If GRACE data are treated with conventional processing methods (using SLR ΔC_20_, GRACE ΔC_21_ and ΔS_21_, PGR model by A *et al*.^18^, and degree-1 via Swenson *et al*.^28^), the estimate becomes 1.86 ± 0.10 mm/yr. Each replacement of the low degree coefficients alters the ocean mass rate by ~0.3 mm/yr, although there is cancellation when all are combined. PGR model choices affect the ocean mass rate only slightly, by about 0.1 mm/yr or less. In different periods, however, the choice of GRACE processing methods leads to more diverse estimates, and the difference amounts to ~0.5 mm/yr depending on the time frame (Table 1). The larger differences are found in estimates for 2005–2013^29,30^. Our new estimate shown in Fig. 3 exhibits an apparent quadratic rather than linear change, so rates of ocean mass increase are larger during later periods. This observation is consistent with acceleration of ice mass loss in Antarctica and Greenland^31^. For comparison with another leakage-corrected solution, we additionally presented an ocean mass rates obtained from CSR GRACE RL05 mascon data for the same period, and it shows lower global mass rates compared with ours. Our estimate of 2.14 mm/yr (about 0.3 mm/yr increase relative to previous studies) would be consistent with a recent estimate of a total sea level rise rate of ~3.5 mm/yr including a steric effect of ~1.1 mm/yr from 2004 to 2015^32^.

**Table 1:** Ocean mass rates in previous studies and this study.

Study	Time period	Ocean mass rate		
		Published	Estimates by FM solution	
			Conventional reduction	Most consistent reduction
Jacob *et al*.^40^	2003.01–2010.12	1.5 ± 0.3^a^	1.4 ± 0.2	1.6 ± 0.2
Johnson *et al*.^44^	2003.01–2012.12	1.8 ± 0.2	1.7 ± 0.1	1.9 ± 0.1
Llovel *et al*.^30^	2005.01–2013.12	2.0 ± 0.1^b^	2.0 ± 0.2	2.5 ± 0.2
Dieng *et al*.^45^	2003.01–2012.12	1.7 ± 0.1^b^	1.7 ± 0.1	1.9 ± 0.1
Dieng *et al*.^29^	2005.01–2013.12	2.0 ± 0.1	2.0 ± 0.2	2.5 ± 0.2
Save *et al*.^9^	2003.01–2014.12	1.5 ± 0.1^c^	1.9 ± 0.1	2.1 ± 0.1
Rietbroek *et al*.^46^	2002.04–2014.06	1.1 ± 0.3		
Dieng *et al*.^32^	2004.01–2015.12	2.2 ± 0.1		
This study	2003.01–2014.12		1.9 ± 0.1	**2**.**1** ± **0**.**1**

Estimates of global ocean mass rates from the recent literatures and this study. We also include some estimates for the same period published earlier, based upon forward modelling solutions of GRACE data reduced by conventional methods (SLR ΔC_20_, PGR correction by the model from A *et al*.^18^, and substitution of degree-1 proposed by Swenson *et al*.^28^) and values for the most consistent methods (alias-corrected CSR GRACE ΔC_20_, ΔC_21_ and ΔS_21_ from polar motion, PGR model from Peltier *et al*.^22^, and degree-1 from this study). Rates are in millimetres per year after the annual cycle has been removed, and uncertainties of FM-based estimates are given at the 2σ (95%) confidence level.

^a^Mass rate estimated by continental ice mass change only. ^b^Uncertainties given at the 1σ confidence level. Otherwise, error estimates are based on the 2σ confidence level. ^c^Estimate based on CSR GRACE RL05 mascon data.

In contrast to global ocean mass rates, values for individual ocean basins vary greatly, and depend significantly on choices of models and methods. Figure 3 (sea level time series for ocean basins including degree-1) shows higher rates in the Southern Hemisphere. Difference in rates among basins are reduced compared to Fig. 1. Presumably the negative mass rate in the Arctic is associated with reduced gravitational attraction as ice melts and mass departs the polar region^33^. Negative mass rates in the Arctic are also evident in Δ*h*_s_ and Δ*h*_g_ rate maps (Fig. 4). Regions near Greenland and West Antarctica, recognized locations of ice mass loss, also show declines, reflecting geoid changes due to declining ice mass in these areas.

Self-consistency between Δ*h*_g_ and Δ*h*_s_ is used in this study to judge various choices in standard GRACE processing steps. However, self-consistency does not necessarily measure the performance of individual choices. For example, the effect of degree-2 values may be affected both by a choice of a substitute estimate and of a PGR model. Self-consistency may result if errors in both offset one another. Thus, our preferred choices in processing methods are not necessarily unique, but instead, reflect the best among those examined.

Further effort is required to improve GRACE processing, but it is encouraging that better self-consistency was generally found using the latest PGR model (*e*.*g*., Peltier *et al*.^22^) and independent (and geophysically well-determined polar motion) degree-2 and order-1 coefficients. This is a sign of progress in models and methods and supports the validity of the self-consistency test. Our GRACE estimates of the global ocean mass rate is consistent with previous studies, but a new contribution of this study is observational evidence of regional ocean mass variations predicted by SLE theory. Significant findings include a decline in Artic ocean mass (about −0.5 mm/yr) and an increase in Southern Hemisphere oceans (about 2.4 mm/yr) exceeding the global average (2.1 mm/yr). These results should support and be enhanced by future research including consideration of altimetry and steric data, and mass redistribution associated with large scale ocean circulation.

## Methods

### Data used in this study

We used RL05 GRACE monthly gravity solutions provided by the Center of Space Research (CSR) and GeoForschungsZentrum in Potsdam (GFZ). These consist of spherical harmonics (SH) coefficients to degree and order 60 for the period from January 2003 to December 2014. Since GRACE data are recognized to have limited ability to estimate degree-2 SH coefficients, they can be replaced by other estimates, including ΔC_20_ coefficients from satellite laser ranging measurements^17^. Further discussion of degree-2 SH coefficients appears below. Contributions of atmospheric surface pressure and ocean bottom pressure have been removed using GRACE Atmospheric and Ocean Dealiasing (AOD) models. ΔC_21_ and ΔS_21_ were also estimated from Earth Orientation Parameters (EOP or polar motion) after correcting effects of pole tide, free wobbles, mantle anelasticity, winds, and ocean currents^34^. Effects associated with relative motion (winds and currents) rather than mass are estimated from ERA-Interim^35^ and GECCO2^36^ numerical models, respectively. The GRACE AOD model was also used to remove the effect of barometric pressure on EOP values of ΔC_21_ and ΔS_21_. An alternative proposed by Wahr *et al*.^16^ (Wahr15) is to estimate ΔC_21_ and ΔS_21_ after correcting for the residual pole tide signal in GRACE ΔC_21_ and ΔS_21_. All ΔC_21_ and ΔS_21_ examined here nominally consider rotational feedback effects^4,16^. After adjusting degree-2 SH coefficients, we removed contributions of Post-Glacial Rebound (PGR) using Stokes coefficients describing a linear in time change of geoid height. We considered three different PGR models including those of A *et al*.^18^ (hereafter, A13), Peltier *et al*.^22^ (Peltier15), and Purcell *et al*.^23^ (Purcell16). The A13 model, the refined version of the model of Paulson *et al*.^37^ (Paulson07), is based on ICE-5G deglaciation history^19^ and the VM2 viscosity profile. Both Peltier15 and Purcell16 are based on the ICE-6G_C ice melting history and VM5a viscosity profile. 500 km Gaussian smoothing and decorrelation filtering were applied to the SH coefficients to suppress noise. SH changes were converted to surface mass change considering contributions of loading and direct gravitational attraction^38^.

### Global forward modelling algorithm

GRACE level 2 data are given as SH expansions truncated at degree and order 60. Spatial filtering needed to suppress noise limits the contribution of high SH degrees. The limited SH range causes gravity change signals to ‘leak’ into adjacent areas. This is particularly evident along the coast, for example near Greenland where gravity signals from melting ice sheets and glaciers leak into adjacent oceans. An iterative algorithm, known as global forward modelling^13^, was developed to address this problem. The algorithm starts with an initial guess of changes in mass distribution on land, then successively refines it until the global distribution of mass change agrees well with GRACE data (in its truncated SH and filtered form), and is uniform over the oceans. In this study, we use a similar approach, with the additional constraint that the distribution of ocean mass is governed by the simplified sea level equation (SLE), and described by Δ*h*_s_. We expect this added constraint to improve estimates of mass change over both oceans and land. The initial model we used here consists of the smoothed land signals of GRACE data and the corresponding mass-conserving uniform mass change over the oceans. In each iteration, the global mean of ocean mass (sea level) was used to judge convergence. We ceased iterations when the difference between two successive solutions was smaller than 1/1000 mm, and solutions mostly converge before 100 iterations. The initial guess of mass change does not sensitively affect the result. At each iteration in the forward modelling, an additional iterative solution for Δ*h*_s_ was necessary; we adopted the fourth iteration of Δ*h*_s_ because it had fully converged. The resolution of the coastline geography is 1 × 1 degree. In the converged FM solution, mass change over land is the updated GRACE land signal, and ocean mass distribution is Δ*h*_s_ which, when integrated over the oceans, is the negative of change over land.

### A second estimate of ocean mass redistribution Δ*h*_g_

The previous section describes how the forward modelling algorithm (FM solution) provides an improved estimate of the distribution of global mass change. Thereafter, we convert mass fields over land from the FM solution to a SH expansion to degree and order 60, and apply 500 km Gaussian smoothing similar to GRACE data reduction. These SH coefficients are converted back to gridded mass fields that, due to their limited SH range and subsequent smoothing, exhibit leakage of terrestrial water storage changes into the oceans. Subtracting this predicted leakage over the oceans from the GRACE data leaves a residual signal over the oceans as a second estimate of ocean mass change that should be free of leakage from (generally larger) signals in adjacent land areas. This leakage-corrected ocean mass estimate, Δ*h*_g_, should be dominated by ocean mass distribution that, on average, conforms to geoid changes, although there will be additional contributions from other sources, including ocean dynamics (ocean currents), and earthquakes.

### Self-consistency test

GRACE data provide two estimates of mass changes over the oceans. Δ*h*_s_ is a prediction of ocean mass distribution and change conforming to the changing geoid, while Δ*h*_g_ refers to GRACE observations over oceans after leakage from land has been subtracted. As two different estimates of the same quantity, they should coincide with one another except that Δ*h*_g_ will retain ocean signals associated with ocean dynamics and other effects. We introduced the term “self-consistency” to describe how well time series of Δ*h*_g_ and Δ*h*_s_ agree. We judge various GRACE processing choices by self-consistency of Δ*h*_g_ and Δ*h*_s_ using root-mean-square (RMS) and linear trend differences of time series over individual ocean basins. Δ*h*_s_ is spatially smoothed in the same manner as GRACE data with a minor effect on linear rates. Ocean basin average time series should suppress differences due to small scale residual errors in Δ*h*_g_. SLE predictions of Δ*h*_s_ are relatively smooth. We selected 6 major ocean basins (North and South Atlantic, North and South Pacific, Indian, and Arctic) and estimated basin average time series for Δ*h*_g_ and Δ*h*_s_.

We first evaluated the self-consistency test using a synthetic GRACE-like data set. Synthetic data set A used RACMO2.3^39^ for surface mass balance over Greenland and Antarctica, GRACE observations^40^ for linear ice mass change associated with ice dynamics over both ice sheets, ERA-interim^35^ for soil moisture, with corresponding gravitationally consistent sea level change over the oceans. To make it similar to GRACE data, we expanded data set A into spherical harmonics, truncated it to SH degree 2 to 60 and applied Gaussian smoothing. By applying forward modelling to this “GRACE-like” data, we obtained corresponding Δ*h*_g_ and Δ*h*_s_ estimates. Time series of Δ*h*_g_ and smoothed Δ*h*_s_ from data set A are almost identical to one another (Supplementary Fig. S1). Furthermore, Δ*h*_g_ (unsmoothed) is almost the same as true sea level changes in the synthetic data (linear trend difference within ~0.01 mm/yr), and estimates of Δ*h*_g_ and smoothed Δ*h*_s_ are nearly identical over the six ocean basins. Although it does not includes effect of errors from the atmosphere, ocean dynamics, and non-surface mass signals such as PGR and pole tide, Supplementary Fig. S1 shows that FM is effective in estimating leakage-free land and ocean signals. We also confirmed that spatial filtering details (*e*.*g*., 300 km or 400 km) and initial mass distribution do not affect the FM outcome.

Several additional error sources, not considered in data set A, may also affect our results. Mass redistribution due to ocean dynamics will be present in GRACE data, but is not included in the SLE prediction. Further, near coastal boundaries, residual ocean dynamics may affect FM leakage prediction. Residual PGR signals unrelated to water and ice mass redistribution may also corrupt the FM solution and affect self-consistency test. Lastly, low-degree GRACE coefficients may contain errors with large spatial scale spurious patterns in Δ*h*_g_. On the other hand, Δ*h*_s_ should not contain such patterns. Considering these three kinds of cases, we created data set B by adding synthetic error contributions to data set A. Ocean dynamic errors were taken from GRACE AOD ocean dynamics model. PGR errors were taken as the difference between A13 and Peltier15. Low-degree SH errors were taken to be ΔC_21_ values of data set A, as a proxy for un-modelled pole tide. The self-consistency test for data set B is shown in Supplementary Fig. S2. There are significant discrepancies between the estimates of Δ*h*_g_ and smoothed Δ*h*_s_ over all ocean basins. Clearly, introduction of these errors in data set B has affected Δ*h*_s_ and Δ*h*_g_ differently, neither reflects true ocean mass changes. This test with data set B shows that neither Δ*h*_s_ nor Δ*h*_g_ provides a good ocean mass change estimates when self-consistency is poor, but it also shows that the self-consistency test should be useful in judging real GRACE data.

We examine sea level change time series for individual ocean basins and multi-basin averages using various combinations of PGR models and methods of adjusting degree-2 SH coefficients. A particular combination is preferred if Δ*h*_g_ and smoothed Δ*h*_s_ have small root-mean-square (RMS) and mass rate differences. PGR models vary depending upon underlying assumptions about ice load geography and melt history, and adopted models of Earth’s elastic and viscous properties. PGR model uncertainty directly affects GRACE mass change estimates. We examined three different PGR models from the recent literature. Adjustments to degree-2 SH coefficients have been routine in GRACE studies because GRACE values are considered unreliable. We examined three different ways to modify SH degree-2 order-1 coefficients and three to adjust degree-2 order-0 coefficients. We considered all possible combinations of PGR models and SH degree-2 adjustments, resulting in 27 different versions of Δ*h*_g_ and Δ*h*_s_. They are listed in Supplementary Table S1. Degree-1 SH coefficients were excluded in the consistency test because they are omitted in GRACE solutions.

Supplementary Figs S3 to S5 illustrate how various processing choices affect the time series. Figure S3 shows results for CSR RL05 GRACE data using the A13 PGR model, SLR ΔC_20_ coefficients, and untouched CSR GRACE ΔC_21_ and ΔS_21_ (combination #7 in the Supplementary Table S1). These have been common choices in recent GRACE studies. Estimates of ocean mass change would likely be poor. Supplementary Fig. S4 shows times series as in Fig. S3, after changing only the PGR model (Paulson07 instead of A13). Paulson07 is the previous version of A13, and it clearly yields reduced self-consistency for most ocean basins. Better self-consistency in Fig. S3 compared to that in Fig. S4 supports the conclusion that A13 is an improvement relative to its predecessor. As another example, because GRACE ΔC_21_ and ΔS_21_ may suffer from an incorrect pole tide correction^16^, substituting polar motion estimates ought to improve self-consistency. Indeed, Supplementary Figs S3 and S5 show greatly improved self-consistency after replacing GRACE ΔC_21_ and ΔS_21_ with polar motion values.

We applied the 27 different processing choice combinations to GRACE data from the two data centres (CSR and GFZ), with results summarized in Supplementary Figs S6 and S7. The horizontal axes identify particular combinations of PGR models and degree-2 adjustments from Supplementary Table S1. The vertical axes display RMS differences (a and c panels in those figures) and trend differences (b and d). Using self-consistency as measured by RMS and trend differences, the preferred methods for CSR data are: to adopt the Peltier15 PGR model; to replace GRACE ΔC_21_ and ΔS_21_ with polar motion estimates, and to retain CSR GRACE ΔC_20_ values after a correction for tidal aliasing (*i*.*e*., #11). In this case, time series of Δ*h*_g_ and smoothed Δ*h*_s_ (Fig. 1) clearly show greater similarity in both trend and amplitude compared to those in Supplementary Fig. S2, indicating that error contributions from ocean dynamics, PGR, and un-modelled degree-2 changes are effectively reduced. The Peltier15 model shows better performance relative to the A13 and Purcell16 model. Although substituting SLR ΔC_20_ for GRACE coefficients has been common, we found that, while this reduces some RMS differences, it increases trend misfits for the CSR solution. Using CSR GRACE ΔC_21_ and ΔS_21_ SH coefficients as published (combinations #1 to #9) leads to poor performance especially in the Atlantic Oceans. Using those from polar motion (#10 to #18) or modified GRACE ΔC_21_ and ΔS_21_ by Wahr15 (#19 to #27) improves the performance, but the former choice leads to better consistency overall. In the Arctic, we find large RMS differences regardless of the combination choice (Supplementary Fig. S7a), suggesting that GRACE estimates for the Arctic are relatively poor.

For GFZ solutions, on the other hand, the preferred methods are: to substitute SLR ΔC_20_ values and modified GFZ GRACE ΔC_21_ and ΔS_21_ by Wahr15 rather than using the GFZ coefficients as published, and to use the Peltier15 model (#26). For GFZ solutions, substitution of SLR ΔC_20_ yields the greatest improvement in self-consistency, and is related to our observation that GFZ ΔC_20_ estimates show peculiar variations relative to others. When GFZ GRACE ΔC_20_ values are retained, both Δ*h*_g_ and Δ*h*_s_ are contaminated, resulting in poor self-consistency. The three choices for ΔC_21_ and ΔS_21_ adjustment had similar effect on self-consistency, since GFZ GRACE values for ΔC_21_ and ΔS_21_ are similar to the alternatives (polar motion and Wahr15). This is not the case for CSR GRACE ΔC_21_ and ΔS_21_.

Overall, better self-consistency is obtained using CSR GRACE solution. Therefore, our sea level estimates associated with ocean mass changes are based on the CSR GRACE data with CSR ΔC_20_ values after correcting tide aliasing, polar motion (EOP) values for ΔC_21_ and ΔS_21_, and Peltier15 PGR model. Nevertheless, both CSR and GFZ solutions provide similar global mass sea level estimates (*i*.*e*., Δ*h*_s_) if the same processing methods are used. This indicates that, at SH degree-3 and above, differences between the two solutions have a relatively small effect on sea level estimates.

### Degree-1 estimates

Degree-1 SH coefficients have been computed from GRACE data in many studies using the method of Swenson *et al*.^28^ (hereafter Swenson08). To estimate the degree-1 coefficients associated with water and ice mass redistribution between land and oceans, Swenson08 incorporated terrestrial surface mass using GRACE data from SH degrees 2 to 60 and uniform ocean mass changes as determined by the negative of total terrestrial mass change. Using this simplified surface mass field, however, leads to errors in degree-1 estimates. The limit SH range of GRACE data produces leakage errors, and a uniform mass change over the oceans does not reflect self-attraction and loading effects. The importance of using Δ*h*_s_ in estimating geocenter motion was also noted in a recent study by Sun *et al*.^41^. That study also considered leakage from land to oceans using a 300 km buffer zone between land and oceans and a limited range of SH coefficients, but found optimum SH truncation using empirical methods based on numerical experiments. These limitations can be effectively addressed using the surface mass field from FM and by imposing a gravitationally consistent ocean mass distribution as estimated in this study. To compare the performance of both approaches, we used synthetic data set A discussed earlier. Changes in degree-1 SH coefficients computed from the synthetic data describe geocenter variations (centre of mass (CM) variations with respect to the centre of figure (CF)) contributed by surface water and ice mass change, which is what we seek. From data set A, we synthesized data using SH degrees 2 to 60 with added simulated GRACE noise^42^. Supplementary Fig. S8 shows the ‘true’ synthetic degree-1 SH coefficients from data set A and estimated degree-1 SH coefficients based on the Swenson08 method. All degree-1 estimates are expressed as geocenter motion along *x*, *y*, and *z*-axes in the CF frame. Geocenter motion computed using the original method of Swenson08 shows relatively large differences compared to the true value, which is probably due to leakage error and the uniform ocean mass assumption. On the other hand, the geocenter estimates from the modified Swenson08 method incorporating leakage corrected mass fields and gravitationally consistent ocean mass changes shows a good match with true values. There remain slight discrepancies between true values and the modified Swenson08 estimates, which is possibly due to the added noise. Without the addition of this noise, each degree-1 value calculated by our method is almost identical to the true. This indicates that the FM method successfully removes spatial leakage, and leads to more accurate estimation of degree-1 terms.

We derived degree-1 estimates from the GRACE data processed by the preferred choices based on the self-consistency test described in the previous section. Estimates of geocenter variation using GRACE data based on our preferred PGR model and degree-2 processing choices, are shown in Supplementary Fig. S9, corresponding to surface mass change neglecting ocean and atmosphere dynamics. Our degree-1 estimates are displayed by a black solid line, and Swenson08 estimates from the GRACE Tellus website are shown in red. Orange and blue solid lines represent estimates from Sun *et al*.^41^ and Wu *et al*.^43^, respectively. Originally, geocenter variations from Wu *et al*.^43^ represents “full” geocenter variations including ocean dynamics and atmospheric pressure, and hence we corrected the geocenter variations using GRACE AOD model to compare with other estimates. Nonetheless, the blue lines differ most among the estimates. On the other hand, we see that estimates represented by red and orange solid lines are largely similar to one another, since both are fundamentally based on the original approach of Swenson08 with GRACE data. Estimates from Sun *et al*.^41^ show more decreasing trend in ΔC_10_ (Δz) but more increasing trend in ΔS_11_ (Δy) relative to the values of Swenson08. In contrast, our ΔC_10_ estimate (black solid line in Supplementary Fig. S9a) shows a more negative trend, indicating more rapid CM motion towards the Southern Hemisphere. With large Northern-Southern Hemisphere differences in land distribution and important polar ice sheet changes, ΔC_10_ ought to be more sensitive (relative to the other two geocenter components) to redistribution of mass from land to oceans. Differences relative to those based on Swenson08 are attributed to corrections for leakage from land to oceans, to related differences in mass change on land, and to our use of Δ*h*_s_ in place of a uniform ocean mass distribution.

## Electronic supplementary material

Supplementary Information

## Data Availability

CSR RL05 GRACE data, SLR ΔC_20_ estimates, AOD model, degree-1 estimates of Swenson08, and PGR model of A13 and Paulson07 are available at GRACE Tellus website (https://grace.jpl.nasa.gov/data/get-data). GFZ RL05 GRACE data are available at GFZ data centre (https://isdc.gfz-potsdam.de/grace-isdc). Degree-1 estimates of Sun *et al*.^41^ can be downloaded from a data centre in TU Delft (https://www.tudelft.nl/citg/over-faculteit/afdelingen/geoscience-remote-sensing/research/research-themes/gravity/models-data/champgracegoce-gravity-models-data/degree-1-and-c20-coefficients), and estimates from Wu *et al*.^43^ can be provided from Dr. Wu upon request. PGR model of Peltier15 can be accessed from Dr. Peltier’s website (http://www.atmosp.physics.utoronto.ca/~peltier/data.php), and Purcell16 model is provided from Supplementary Information of their paper. RACMO2.3 data are available upon request in Institute for Marine and Atmospheric Research, Utrecht University (https://www.projects.science.uu.nl/iceclimate/models/greenland.php). ERA-interim data is provided via ECMWF app (http://apps.ecmwf.int/datasets/data/interim-full-aily/levtype=sfc/). Additional data related to this paper can be requested from the authors.
