# Supplementary material for: Global sea level change signatures observed by GRACE satellite gravimetry
Source: Sci Rep. 2018 Sep 10;8:13519. doi: 10.1038/s41598-018-31972-8 (PMC6131218; doi:10.1038/s41598-018-31972-8)
Supplement: Supplementary file 1 — Supplementary Information [file 41598_2018_31972_MOESM1_ESM.pdf]

Supplementary Information for

Global sea level change signatures observed by GRACE satellite gravimetry

By Taehwan Jeon, Ki-Weon Seo*, Kookhyoun Youm, Jianli Chen, Clark R. Wilson

It includes

- Supplementary Figures from S1 to S9
- Supplementary Table S1

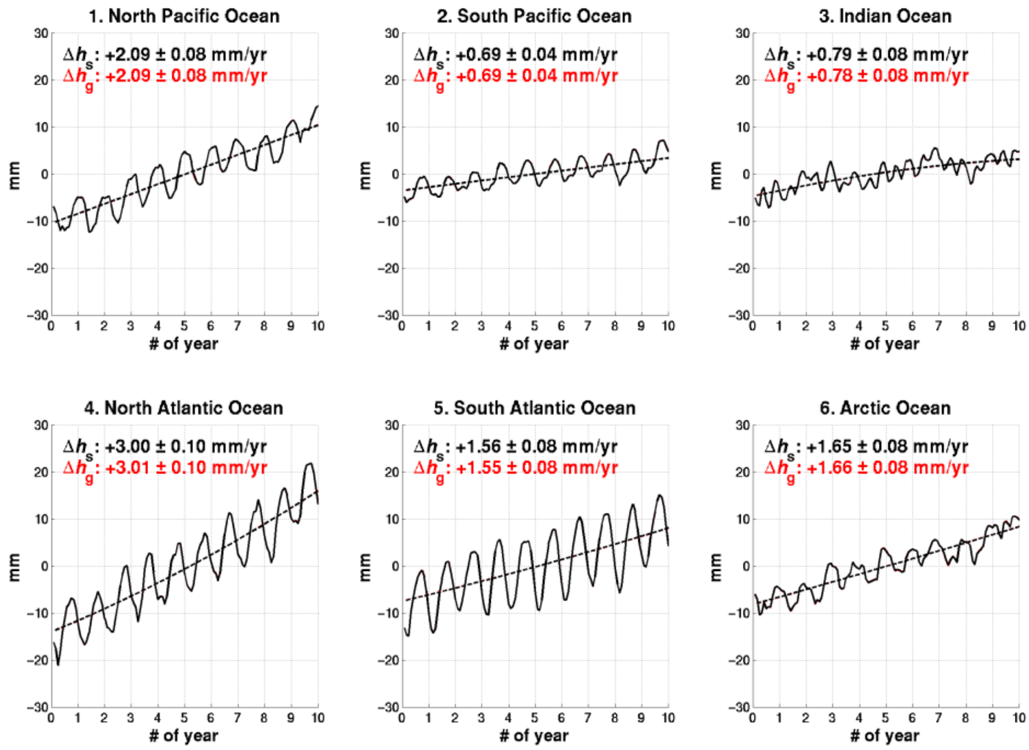

Supplementary Fig. S1. Self-consistency of synthetic data set A. Smoothed Δh_s (black solid line) and Δh_g (red solid line) of sea level due to ocean mass variations in millimeters over 6 ocean basins calculated from 10-year-long monthly synthetic data. The data used here is synthetic data set A described in the text. Two curves completely overlap with each other. Trend estimates are from a second-order least squares polynomial fit to time series after removing annual variations.

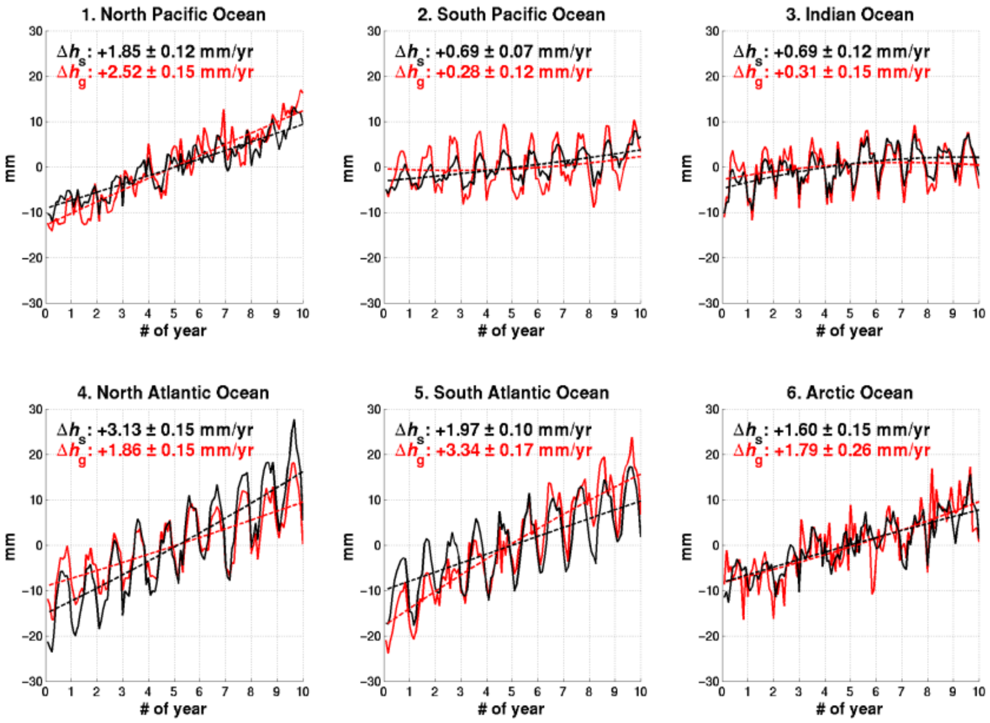

Supplementary Fig. S2. Self-consistency of synthetic data set B. Similar to Supplementary Fig. S1 except using synthetic data set B described in the text.

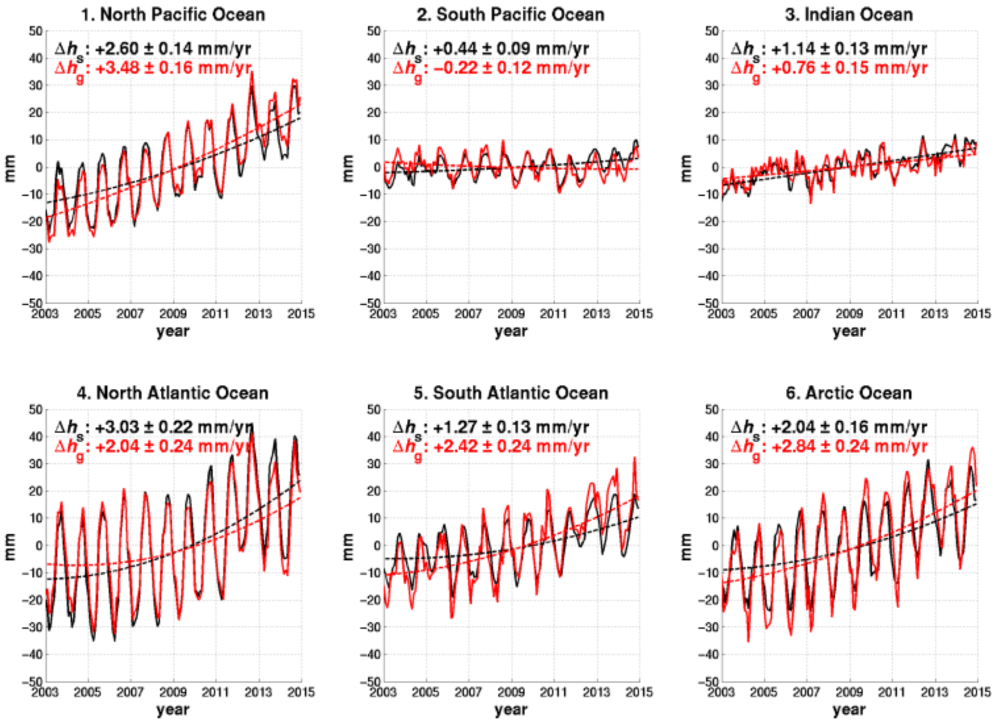

Supplementary Fig. S3. Self-consistency of GRACE data post-processed by conventional choices. Smoothed Δh_s (black solid line) and Δh_g (red solid line) of sea level due to ocean mass variations in millimeters over 6 ocean basins for January 2003 to December 2014. Δh_s and Δh_g are computed using CSR RL05 GRACE solutions with reduction of PGR signals via a model of A *et al.*[18], substitution of SLR ΔC_{20} coefficients, and no adjustments to GRACE ΔC_{21} and ΔS_{21}. Trend estimates are from a second-order least squares polynomial fit to time series after removing annual variations. Differences in trends indicate lack of consistency for this combination.

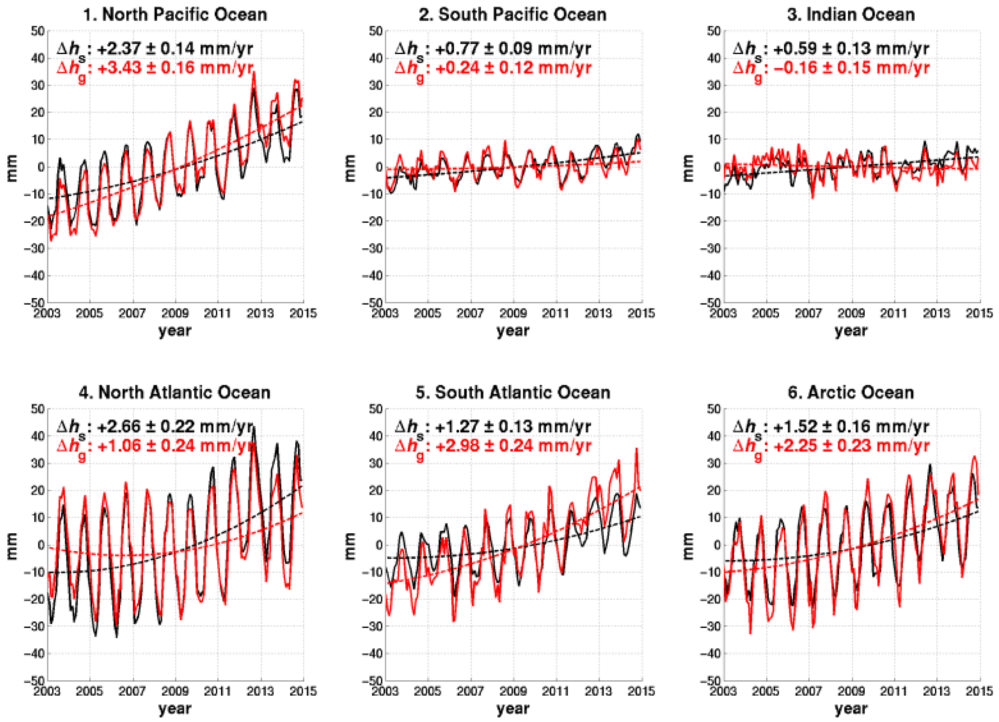

Supplementary Fig. S4. Self-consistency of GRACE data with different PGR reduction. Similar to Supplementary Fig. S3 except that PGR model of Paulson *et al.*[41] was used instead of A *et al.*[18]. The discrepancies between two curves slightly increased compared to the result in Supplementary Fig. S3.

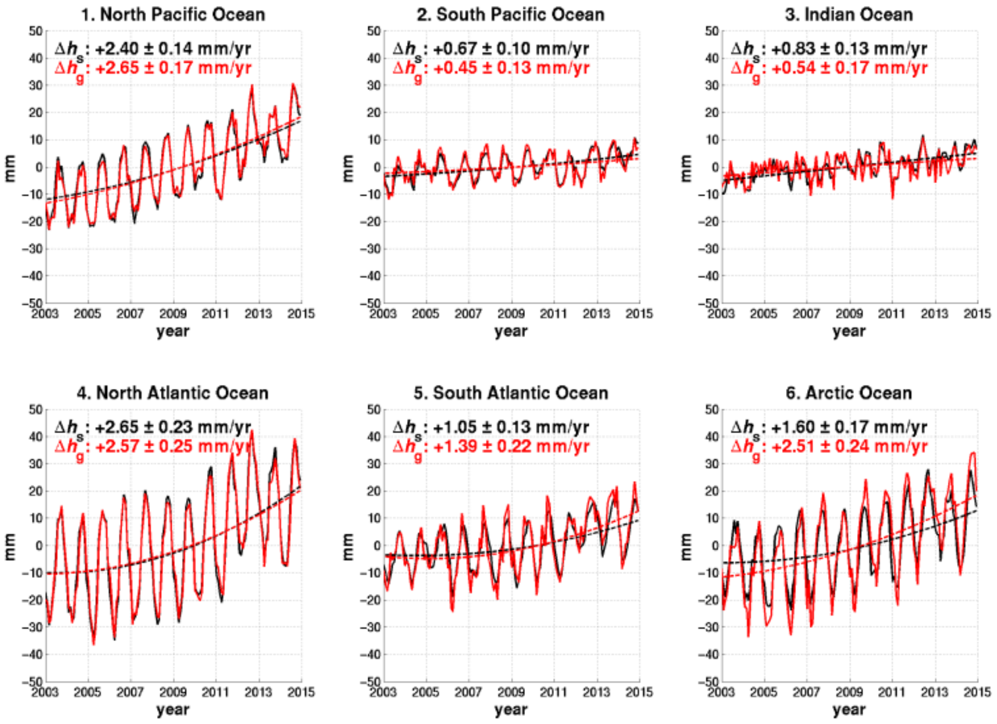

Supplementary Fig. S5. Self-consistency of GRACE data with EOP C_{21} and S_{21}. Similar to Supplementary Fig. S3 except that ΔC_{21} and ΔS_{21} from polar motion have been substituted for GRACE values. The result shows greatly improved consistency in trends for most ocean basins, except the Arctic.

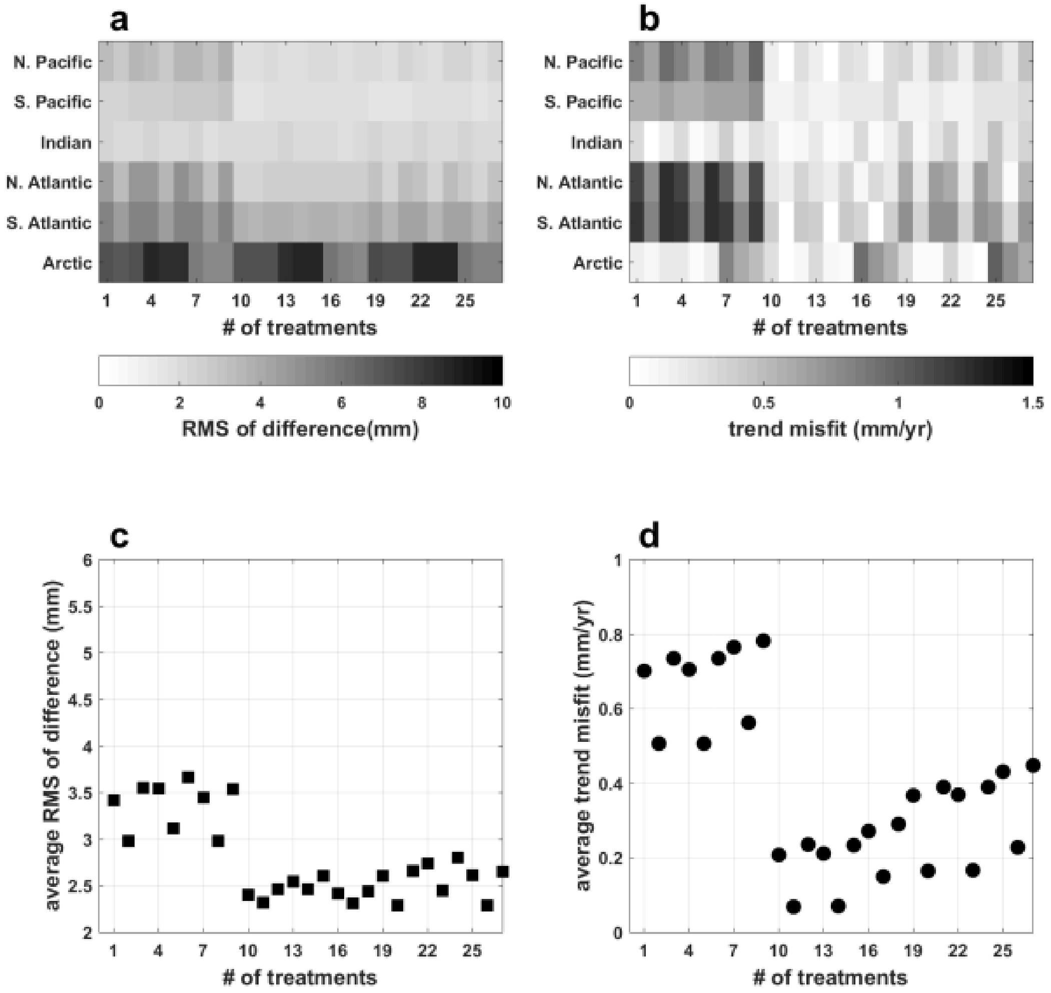

Supplementary Fig. S6. RMS differences and trend misfits for examined post-processing combinations for CSR GRACE data from 2003 to 2014. (a) root-mean-square (RMS) of differences between Δh_g and smoothed Δh_s for individual ocean basins. (**b**) Trend differences between both time series for individual ocean basins. **(c)** RMS difference for an area-weighted average of all basins. **(d)** Trend differences for an area-weighted average of all basins. The *x*-axis corresponds to combinations (treatments) of PGR models and degree-2 adjustments listed in Supplementary Table S1.

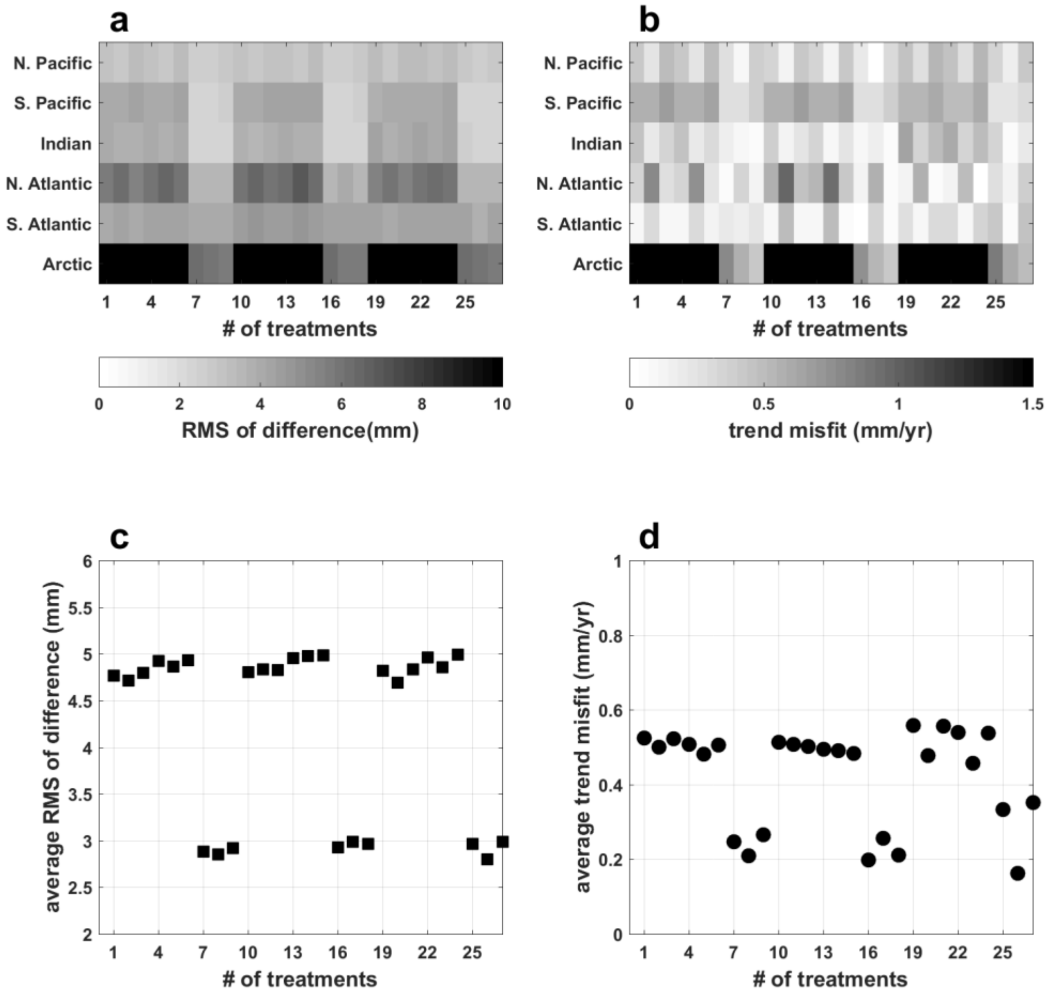

Supplementary Fig. S7. RMS differences and trend misfits for examined post-processing combinations for GFZ GRACE data from 2003 to 2014. (a) root-mean-square (RMS) of differences between Δh_g and smoothed Δh_s for individual ocean basins. (**b)** Trend differences between both time series for individual ocean basins. **(c)** RMS difference for an area-weighted average of all basins. **(d)** Trend differences for an area-weighted average of all basins. The *x*-axis corresponds to combinations (treatments) of PGR models and degree-2 adjustments listed in Supplementary Table S1.

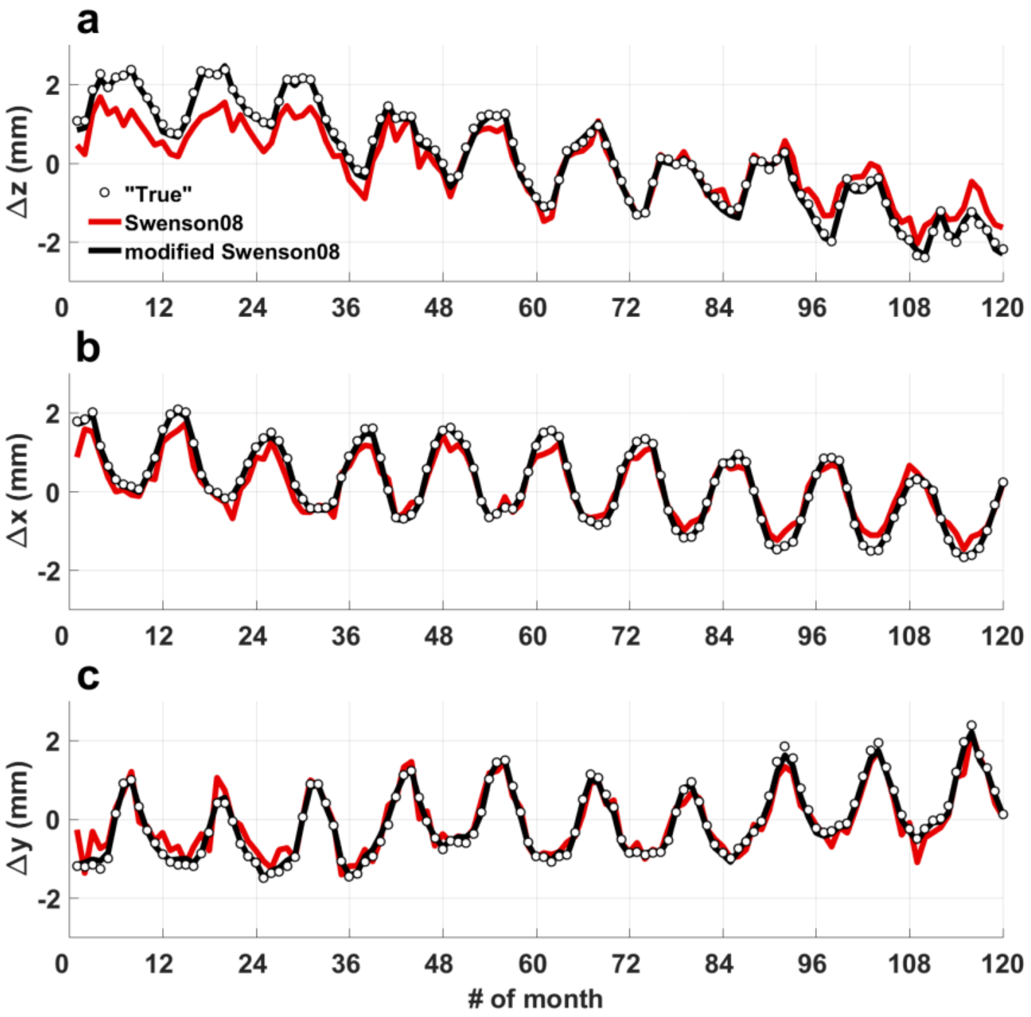

Supplementary Fig. S8. Synthetic test for degree-1. Geocenter (degree-1) estimates for Δz **(a)**, Δx **(b)**, and Δy **(c)** by using 120-month synthetic data set described in the text. The white dots represent true geocenter motion from the synthetic data. Geocenter estimates based on the Swenson08 method (red solid lines) show large discrepancies compared to the true ones. Alternative geocenter estimates using the modified Swenson08 method (black solid lines) yield nearly identical values to the true.

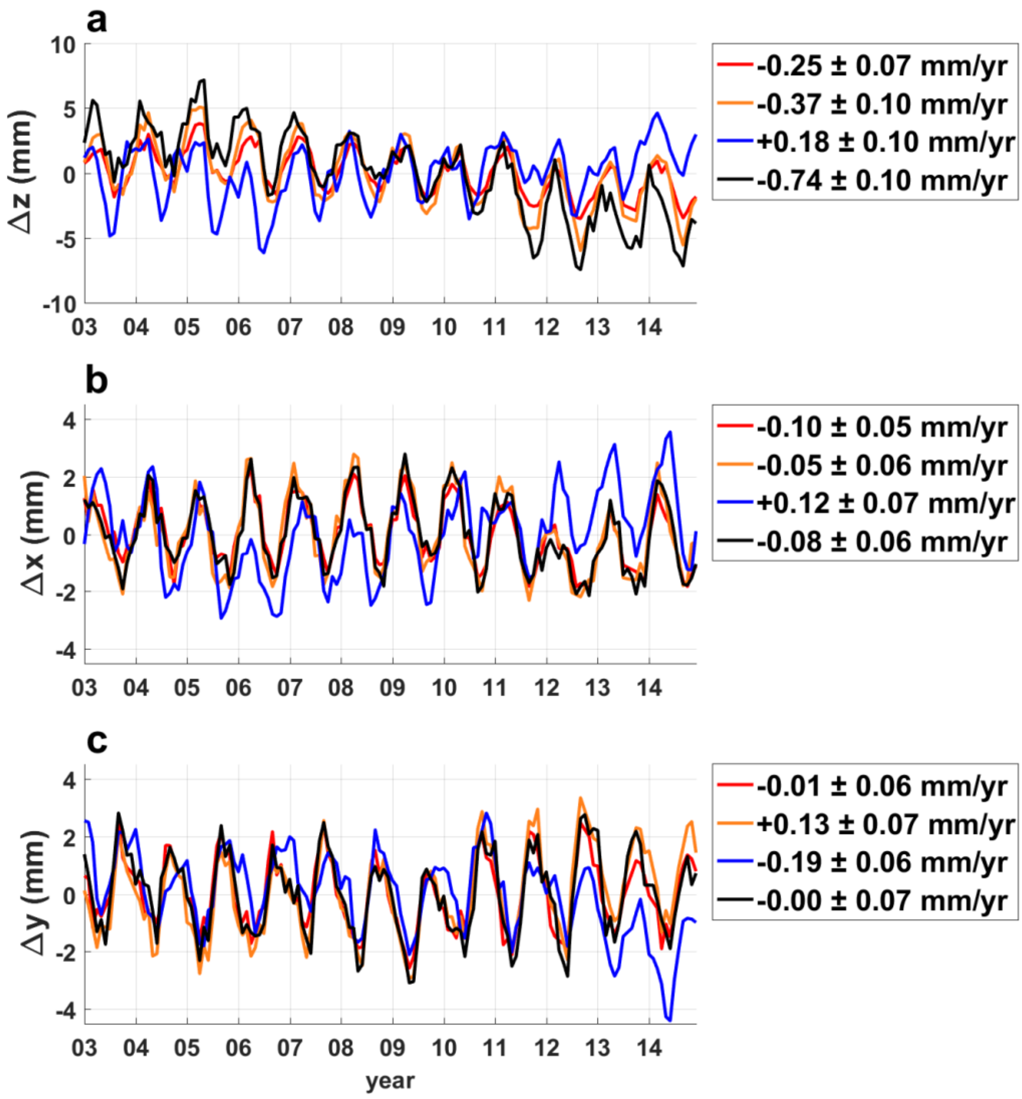

Supplementary Fig. S9. Degree-1 estimates. Estimates of geocenter motion of Δz (**a**), Δx (**b**), and Δy (**c**) due to surface mass change, neglecting atmosphere and ocean dynamics, from 2003 to 2014. Red solid lines are estimates of Swenson *et al.*[28] from GRACE Tellus website, and orange solid lines are those from Sun *et al.*[44], very similar to Swenson08. Blue solid lines represent estimates from Wu *et al.*[46]. Black solid lines are estimates from this study, using GRACE data with the preferred PGR model and degree-2 substitutions as described in the text.

Supplementary Table S1. List of 27 post-processing combinations for GRACE data. We examined 27 combinations of degree-2 SH coefficient adjustment methods and PGR models. For the degree-2 order-0 coefficient, choices are to use satellite laser ranging (SLR) values, GRACE (GR) values, or GRACE values with tidal aliasing corrections for S_2 and K_2 (GRc) as described in the text. Further, three choices for degree-2 and order-1 SH coefficients are examined: GRACE (GR), polar motion (Earth Orientation Parameter denoted as EOP), and GRACE values modified by the method of Wahr *et al.*[16] (Wahr15). The three PGR models of A *et al.*[18] (A13), Peltier *et al.*[22] (Peltier15), and Purcell *et al.*[23] (Purcell16) are listed here.

#	C_{20}	C_{21} & S_{21}	PGR
1	GRc	GR	A13
2	GRc	GR	Peltier15
3	GRc	GR	Purcell16
4	GR	GR	A13
5	GR	GR	Peltier15
6	GR	GR	Purcell16
7	SLR	GR	A13
8	SLR	GR	Peltier15
9	SLR	GR	Purcell16
10	GRc	EOP	A13
11	GRc	EOP	Peltier15
12	GRc	EOP	Purcell16
13	GR	EOP	A13
14	GR	EOP	Peltier15
15	GR	EOP	Purcell16
16	SLR	EOP	A13
17	SLR	EOP	Peltier15
18	SLR	EOP	Purcell16
19	GRc	Wahr15	A13
20	GRc	Wahr15	Peltier15
21	GRc	Wahr15	Purcell16
22	GR	Wahr15	A13
23	GR	Wahr15	Peltier15
24	GR	Wahr15	Purcell16
25	SLR	Wahr15	A13
26	SLR	Wahr15	Peltier15
27	SLR	Wahr15	Purcell16

References cited for Supplementary Information

16 Wahr, J., Nerem, R. S. & Bettadpur, S. V. The pole tide and its effect on GRACE time-variable gravity measurements: Implications for estimates of surface mass variations. J. Geophys. Res. Solid Earth 120, 4597-4615 (2015).

18 A, G., Wahr, J. & Zhong, S. Computations of the viscoelastic response of a 3-D compressible Earth to surface loading: an application to Glacial Isostatic Adjustment in Antarctica and Canada. Geophys. J. Int. 192, 557-572 (2013).

22 Peltier, W. R., Argus, D. F. & Drummond, R. Space geodesy constrains ice age terminal deglaciation: The global ICE-6G_C (VM5a) model. J. Geophys. Res. Solid Earth 120, 450-487 (2015).

23 Purcell, A., Tregoning, P. & Dehecq, A. An assessment of the ICE6G_C(VM5a) glacial isostatic adjustment model. J. Geophys. Res. Solid Earth 121, 3939-3950, doi:10.1002/2015JB012742 (2016).

28 Swenson, S., Chambers, D. & Wahr, J. Estimating geocenter variations from a combination of GRACE and ocean model output. J. Geophys. Res. Solid Earth 113, B08410 (2008).

41 Paulson, A., Zhong, S. & Wahr, J. Inference of mantle viscosity from GRACE and relative sea level data. Geophys. J. Int. 171, 497-508 (2007).

44 Sun, Y., Riva, R. & Ditmar, P. Optimizing estimates of annual variations and trends in geocenter motion and J2 from a combination of GRACE data and geophysical models. J. Geophys. Res. Solid Earth 121, 8352-8370 (2016).

46 Wu, X., Kusche, J. & Landerer, F. W. A new unified approach to determine geocentre motion using space geodetic and GRACE gravity data. Geophys. J. Int. 209, 1398-1402, doi:10.1093/gji/ggx086 (2017).
